# Marine-Derived Lead Fascaplysin: Pharmacological Activity, Total Synthesis, and Structural Modification

**DOI:** 10.3390/md21040226

**Published:** 2023-03-31

**Authors:** Chao Wang, Siyuan Wang, Haonan Li, Yonglian Hou, Hao Cao, Huiming Hua, Dahong Li

**Affiliations:** 1 Key Laboratory of Structure-Based Drug Design & Discovery, Ministry of Education, and School of Traditional Chinese Materia Medica, Shenyang Pharmaceutical University, 103 Wenhua Road, Shenyang 110016, China; 2 College of Pharmacy, Shenzhen Technology University, Shenzhen 518118, China; 3 School of Life Science and Biopharmaceutics, Shenyang Pharmaceutical University, Shenyang 110016, China

**Keywords:** marine alkaloid, fascaplysin, synthetic chemistry, anti-tumor

## Abstract

Fascaplysin is a planar structure pentacyclic alkaloid isolated from sponges, which can effectively induce the apoptosis of cancer cells. In addition, fascaplysin has diverse biological activities, such as antibacterial, anti-tumor, anti-plasmodium, etc. Unfortunately, the planar structure of fascaplysin can be inserted into DNA and such interaction also limits the further application of fascaplysin, necessitating its structural modification. In this review, the biological activity, total synthesis and structural modification of fascaplysin will be summarized, which will provide useful information for pharmaceutical researchers interested in the exploration of marine alkaloids and for the betterment of fascaplysin in particular.

## 1. Introduction

Since entering the 21st century, the potential of unique natural products from marine as candidate drugs has been widely recognized, and significant advancement has been made in the clinical development of marine-derived medicines [1,2,3,4]. Currently, the world has increasingly acknowledged the promise of marine natural ingredients as drug candidates, and the field is under vigorous development recently [5,6,7,8,9]. After decades of exploration, a growing number of marine alkaloids have been discovered, mainly from invertebrates such as sponges, tunicates, bacteria, as well as marine bacteria and cyanobacteria. Due to the complex structure of metabolites, their structural transformation, stereochemistry, synthesis, and pharmacology received extensive attention from many disciplines [10,11,12,13]. These marine alkaloids are structurally abundant, including β-carboline alkaloids, bromine and iodine alkaloids, monoamine alkaloids, diterpene alkaloids, indole alkaloids, pyridoacridine alkaloids, polycyclic guanidine alkaloids, pyrrole alkaloids, quinoline alkaloids, and piperidine alkaloids [14,15,16,17,18]. Marine alkaloids also have a variety of pharmacologically activities, including diverse antimicrobial activity [19], anti-inflammatory activity [20], anti-tumor activity [21], anti-plasmodium activity, etc. [22,23].

**Figure 1:**
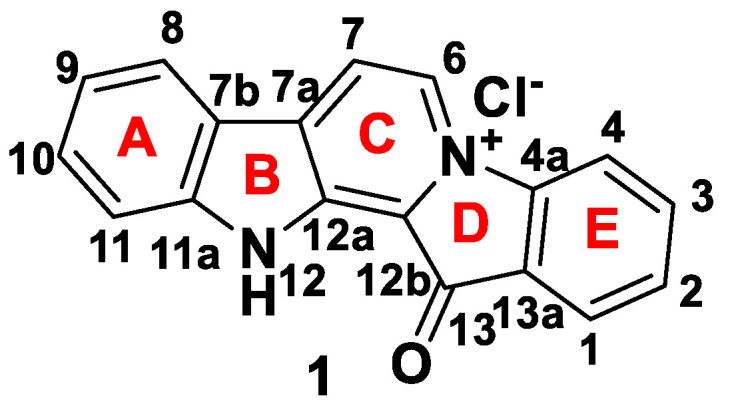
Chemical structure and numbering of fascaplysin.

Fascaplysin (**1**, Figure 1), 13-oxo-12,13-dihydropyrido[1,2-a:3,4-b′]diindol-5-ium chloride, was first isolated from the sponge in 1988 and belongs to the alkaloid analog [17]. In terms of structure, fascaplysin has a special plane structure that can be intercalated in DNA. As for pharmacological effects, fascaplysin also exhibits promising biological activities, such as antibacterial activity, anti-tumor activity, anti-plasmodium activity, etc. Up until now, although there are many studies on fascaplysin and its derivatives, little of the literature systematically summarizes fascaplysin. Therefore, the synthetic chemistry and the pharmacological activity of fascaplysin and its structurally modified derivatives will be introduced in this review.

## 2. Pharmacological Activity

The pharmacological activities of fascaplysin include analgesic activity, antithrombotic activity, anti-Alzheimer activity, anti-plasmodial activity, and anti-tumor effect. These can be classified as follows:

### 2.1. Anti-Tumor Effects 

#### 2.1.1. Anti-Tumor Mechanism 

Fascaplysin also shows good inhibitory effects on multifarious tumor cells. The main mechanisms of its anti-tumor effects are identified as: (i) a selective inhibition of cyclin-dependent kinase 4 (CDK4), which regulates the G0/S and G1/S checkpoint of the cell cycle. The final initiation of apoptosis requires the activation of a series of caspase proteases, in which the up-regulation of DR5 expression by fascaplysin may activate the receptor-mediated by TRAIL apoptotic signal, and hence, the onset of programmed cell death via fascaplysin [24,25,26,27]. (ii) Antiangiogenic, fascaplysin inhibits the expression and excretion of vascular endothelial growth factor (VEGF), and destroys angiogenesis to achieve anti-tumor effect [26,27]; and (iii) the consecutive induction of apoptosis [28,29,30,31]..

**Figure 2:**
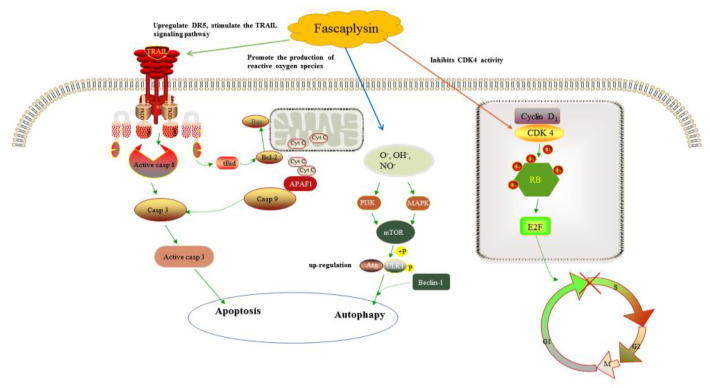
Anti-tumor mechanisms of fascaplysin.

The mechanism of fascaplysin is shown in Figure 2.

#### 2.1.2. Inhibition of Human Alveolar Rhabdomyosarcoma Cells

Chen et al. found that fascaplysin led to the inhibition of the transcriptional activity of PAX3-FOXO1 in alveolar rhabdomyosarcoma (ARMS) cell line Rh30 through inhibiting CDK4, which is consistent with the finding that activation of CDK4 enhances the activity of PAX3-FOXO1. More than 80% of ARMS contain PAX3-FOXO1, which is a fusion transcription factor. Inhibiting the activity of CDK4 may be a promising way to develop therapeutic methods for ARMS [32].

#### 2.1.3. Inhibition of Leukemia Cells

Studies by Bhushan et al. showed that fascaplysin induced caspase arbitrated autophagy and apoptosis through PI3K/Akt/mTOR signaling pathway. They found that fascaplysin induced HL-60 cell cycle stasis in the sub G1 phase. The inhibitory effect of fascaplysin on HL-60 cell proliferation was potent with IC_50_ values of 1.3 μM, 1 μM, 0.7 μM, and 0.5 μM for 6 h, 12 h, 24 h, and 48 h. With the increase of fascaplysin concentration, the LC3-II autophagic protein expression level increased. Cells treated with fascaplysin showed an increased expression of beclin 1 (BECN 1) and ATG7, which are considered as key autophagic proteins, demonstrating that fascaplysin was capable of robust autophagy induction effect in HL-60 cells. The PI3K/Akt/mTOR signal path is important for cell growth. Fascaplysin inhibited all the key proteins in this signaling pathway, including p110á, pAKT (S473), pAKT (T308), p-mTOR, pP70S6K, raptor, and rictor at 0.5 μM. Because fascaplysin has strong inhibitory effects on Akt and p70S6K, the two proteins are completely suppressed in HL-60 cells. Results confirmed the partial dependence of autophagy and apoptosis induced by fascaplysin on the inhibition of the PI3K/Akt pathway [26]. 

#### 2.1.4. Inhibition of Liver Cancer Cells and Human Venous Endothelial Cells

According to the research of Chen et al., fascaplysin showed an obvious regulating effect on the TNF and TNF receptor superfamily in human umbilical vein endothelial cells (HUVEC) and human hepatocarcinoma cells Bel-7402, and the expression level of cleaved caspase-9 and active caspase-3 was improved while the expression of procaspase-8 and Bid was reduced. Fascaplysin increases cell sensitivity to apoptosis, which is induced by ligands associated with tumor necrosis. TRAIL R2/FC chimera has a significant blocking effect on this apoptosis. The results showed that fascaplysin could promote apoptosis, which was mainly achieved by increasing the expression level of DR5 and activating TRAIL pathway. At the same time, other results showed that the anti-proliferation effect of fascaplysin on Hela cells was not to block the cell cycle, but to induce cell apoptosis through extrinsic and mitochondrial pathways [33].

Meng et al. illustrated the key points of the effects of fascaplysin on vascular endothelial cells (VECs) and provided insights into the role of fascaplysin in cancer therapy. It found that fascaplysin caused autophagy of vascular endothelial cells. Inhibition of autophagy by using an inhibitor (3-methyladenine) or RNA interference of an essential autophagy gene (ATG5) increased the mortality of HUVECs, and at the same time, increased the anti-angiogenic activity of fascaplysin. For tumor cells, high concentrations of ROS caused oxidative damage to proteins, DNA, and lipids, and thus induced apoptosis and autophagy. They also found that fascaplysin significantly increased the levels of p8 protein and ROS, and reduced the mitochondrial membrane potential. Therefore, mitochondria released cytochrome c and apoptosis-inducing factor (AIF) into the cytoplasm through the hole, and then triggered the caspase cascade reaction to damage DNA and caused tumor cell apoptosis. These results indicated that fascaplysin activated autophagy mainly through ROS and p8 in VECs and took it as a kind of cytoprotective response [34].

Yan et al. found that fascaplysin inhibited VEGF in the process of anti-angiogenesis, arrested cell cycle at G1 phase, and induced apoptosis. The apoptosis was also confirmed by the detection of active caspase-3, Bax, Bcl-2, procaspase-8, and Bid. It was also noted that the increase of the ratio of Bax/Bcl-2 indicated that the apoptosis of HUVEC cells might be related to the mitochondria pathway [35]. Lin et al. confirmed that fascaplysin selectively inhibited endothelial cell proliferation toward tumor cells in low concentrations and inhibited the secretion and expression of VEGF in Bel-7402 cells [36].

#### 2.1.5. Inhibition of Melanoma

Lim et al. indicated that compared to two distinct and specific CDK4 inhibitors, PD0332991 and LY2835219, fascaplysin sensitively inhibited the proliferation of A375 malignant melanoma cells, inhibited TRKA and VEGFR2, and down-regulated survivin and HIF-1α, which prevented the formation of tumors. Fascaplysin shows a possible therapeutic strategy for the management of multiple types of solid cancer [37]. Fascaplysin caused apoptosis and decreased clonogenic development. It achieved 99.2% growth inhibition in Sk-Mel-28 cells and 98.0% growth inhibition in Sk-Mel-2 cells. The results indicated that fascaplysin induced the increase in the sub G1 fraction, which was caused by apoptosis [38].

#### 2.1.6. Inhibition of Small Cell Lung Cancer Cells

Hamilton et al. investigated the cytotoxicity of fascaplysin to a panel of small cell lung cancer (SCLC) cell lines and the synergistic effect of fascaplysin and chemotherapy drugs. High cytotoxicity was found for fascaplysin against SCLC cells, and it was reported to promote G0/G1 cell cycle arrest and S-phase cell cycle induction, respectively. Fascaplysin produced ROS and caused the NCI-H417, a drug-resistant SCLC cell line, to undergo apoptotic. In addition, the synergy among fascaplysin and topoisomerase I and 10-hydroxy-camptothecin was significant. The poly ADP-ribose polymerase 1 (PARP1) inhibitor BYK204165 antagonized the cytotoxic effects of fascaplysin, indicating that DNA repair was involved in its anti-tumor effects. According to its high cytotoxicity from multiple pathways, which affected topoisomerase I, the integrity of DNA, and the generation of ROS, fascaplysin seemed to be appropriate for treating SCLC [39].

In primary non-small cell lung cancer (NSCLC) cells, SCLC cells, and SCLC circulating tumor cell lines (CTCs), fascaplysin showed high cytotoxicity to SCLC cell lines (mean IC_50_ = 0.89 μM), to CTCs as single cells, and in the form of tumorospheres (mean IC_50_ = 0.57 μM). For NSCLC cell lines, IC_50_ of fascaplysin was 1.15 μM. Fascaplysin revealed at least an additive cytotoxic effect with cisplatin [40]. Hamilton et al. further found that the active forms of fascaplysin and cisplatin were different. Additionally, fascaplysin increased the anti-tumor efficacy of the EGFR tyrosine kinase inhibitor (TKI) Afatinib by a factor of two. Differences in effects between fascaplysin and cisplatin were confirmed by the CI (combination indices) values of interacting with Chk1/2 inhibitor AZD7762. Protein phosphorylation results revealed that the stress response mediators in H1299 NSCLC cells were hypophosphorylated, while Akt1/2/3 and ERK1/2 were hyperphosphorylated. The combination of fascaplysin and Afatinib further increased cytotoxicity against pleural primary NSCLC lines [41].

Luo et al. found that fascaplysin inhibited migration by regulating the Wnt/β-catenin signaling pathway and reversing the epithelial-mesenchymal transition phenotype, and inhibited the anti-NSCLC effects, mainly by inducing ferroptosis and apoptosis [42].

#### 2.1.7. Inhibition of Ovarian Cancer Cells

In ovarian carcinoma cell lines, fascaplysin greatly increased cell apoptosis and reduced the migration and invasion ability of ovarian cancer cell lines A2780 and OVCAR3. Fascaplysin treatment also reduced the expression levels of cyclin D1, CDK4, Bcl-2 and VEGFA, demonstrating that fascaplysin showed anti-tumor activity on ovarian cancer cell lines by inhibiting CDK4 [43].

#### 2.1.8. Inhibition of Other Tumor Cells

The prognosis for patients with glioblastoma multiforme, an invasive malignant glial brain tumor, is dismal [44]. Igor et al. found that 0.5 μM fascaplysin showed strong cytotoxicity to the cells of glial tumors (C6), and the increased concentration could kill glioma cells, which was more effective than temozolomide [45]. Moreover, fascaplysin was tested for cytotoxicity against a prostate cancer cell line (LNCaP) with an IC_50_ value of 0.54 μM by Khokhar et al. [46].

#### 2.1.9. Increasing the Anticancer Effect of Other Drugs

Lim et al. found that fascaplysin could increase the phosphorylation level of protein kinase B (PKB) and adenosine monophosphate-activated protein kinase (AMPK). With its role in promoting survival or anti-apoptosis in cancer, it is regarded as a therapeutic target for cancer. Fascaplysin enhanced the anticancer effects of a selective AMPK inhibitor and improved the curative effect of MTX on cancer [47].

### 2.2. Analgesic Activity

The analgesic effect of endorphins, the endogenous peptide agonists for opioid receptors (ORs), is significant. The tolerance and dependence of the effect are very small. Endorphins involve both G-protein and *β*-arrestin pathways as “balanced” agonists. At the *μ*-OR, fascaplysin demonstrated activity with an EC_50_ value of 6.3 μM. Importantly, fascaplysin functioned as a balanced agonist, favoring endorphin-like receptor endocytosis while also boosting G-protein signaling and *β*-arrestin recruitment. These results showed that in the field of neuroscience, fascaplysin has great potential as a leading molecule for innovation as a therapeutic target [48].

### 2.3. Anti-Thrombotic Activity 

When the concentration of fascaplysin was 10 µM, it significantly reduced ERK phosphorylation, illustrating that fascaplysin reduced the activity of PI3K in platelets. Fascaplysin markedly blocked the activation of GPIIb/IIIa, as indicated by decreased PAC-1 binding levels after PAR-1-AP, ADP or PMA stimulation. Fascaplysin was also noted to reduce platelet aggregation and PLA formation. The PMA-induced interaction of platelets with monocytes or granulocytes was significantly reduced in the presence of fascaplysin. Fascaplysin-treated mice showed a considerably longer full vascular occlusion duration. Fascaplysin also prolonged the tail-vein bleeding time. Thus, the anti-thrombotic effect of fascaplysin could be verified [49].

### 2.4. Anti-Alzheimer Activity 

Acetylcholinesterase (AChE) is an enzyme in charge of the death of neurons in Alzheimer’s disease. Fascaplysin inhibited AChE is non-competitive, with IC_50_ and Ki values of 1.49 and 2.28 μM, respectively, and with a 60-fold selectivity for AChE versus butyrylcholinesterase. Fascaplysin exhibited promising P-gp activation together with AChE inhibition at 1 mM, with good medication safety (LS-180: IC_50_ > 10 μM, hGF: 4 μM), clearly indicating their promise for the development as an anti-Alzheimer agent [50].

### 2.5. Anti-Plasmodial Activity

In an early study, fascaplysin inhibited *P. falciparum* strain K1 with an IC_50_ value of 50 ng/mL and *P. falciparum* strain NF54 with an IC_50_ value of 34 ng/mL, which showed that fascaplysin was a potent in vitro inhibitor of chloroquine-susceptible (NF54) and chloroquine-resistant *P. falciparum* strains [51]. Due to the potent anti-plasmodial activity, fascaplysin demonstrates the potential to be a leading structure.

## 3. Total Synthesis 

Although fascaplysin has an extensive bioactive spectrum, its further study has been hampered by the limited amount of compounds isolated from marine microorganism sources. As a result, many research groups have devoted their effort to total synthesis [52]. Bharate et al. summarized the total synthesis of fascaplysin reported in the literature up to 2012 [53]. Here, we update the literature reports on total synthesis from 2013 to 2022.

**Scheme 1:**
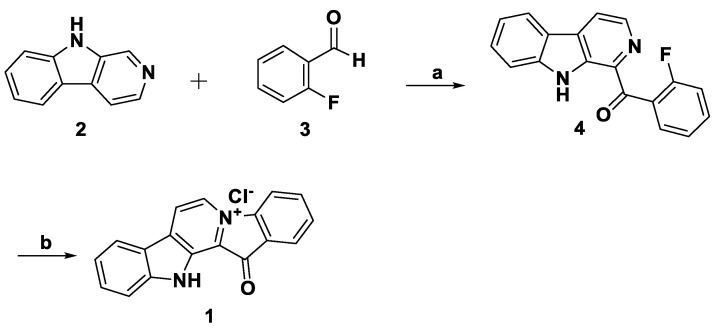
Synthetic method 1. Reagents and conditions: (a) 70% *t*-BuOOH, MW, 10 W, CF_3_COOH, 40 min, several times; (b) Py·HCl, 200–220 ℃, 40 min.

Zhidkov et al. developed a short and efficient method for the synthesis of fascaplysin (Scheme 1) [54]. Microwave irradiation of **2**, **3**, and *t*-BuOOH mixtures in acetic acid gave rise to a yield of 65% for target compound **4**. Heating of **4** with pyridinium chloride at 200–220 ℃ to produce fascaplysin.

**Scheme 2:**
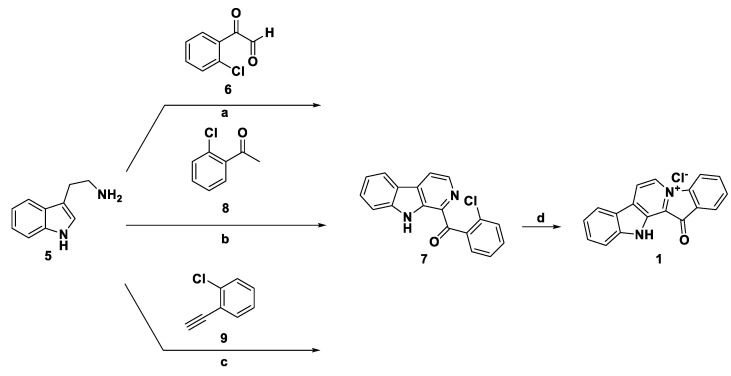
Synthetic methods 2–4. Reagents and conditions: (a) 10% Pd/C (20 mol%), AcOH, reflux, 3 h, 85%; (b) I_2_, DMSO, 90 ℃; (c) I_2_, DMSO, 100 ℃, 4 h; (d) 220 ℃, 20 min.

Bharate et al. reported a two-step method to synthesize fascaplysin (Scheme 2) [55]. Tryptamine (**5**) reacted with glyoxal (**6**) under microwave conditions to form *β*-carboline (**7**) in 80% yield. Heating **7** at 220 ℃ for 20 min resulted in a ring-closure product of fascaplysin. The tandem dehydrative condensation between ortho-halo-substituted glyoxal and tryptamine followed by dehydrogenation is a crucial step in the current method.

A novel reaction of tryptamine and 2-oxoaldehydes under Pictet–Spengler was investigated and fascaplysin was successfully synthesized (Scheme 2) [56]. The reaction of **5** with 2-chloro-acetophenone (**8**) under optimal conditions formed **7** (75%), resulting in fascaplysin when further heated at 220 ℃. Acetophenones/styrenes could easily convert into the necessary products, regardless of how electron-rich and electron-deficient they were. Styrenes produced lower yields than acetophenones, which was why acetophenones were found to be more effective. Furthermore, the effectiveness of the reaction remained impervious to substituents at various positions of the arene group or by the nature of their electronic states. 

The reaction of **5** with 2-chloro-phenylacetylene (**9**) under optimal conditions formed compound **7** (83%). Performing the reaction under inert atmosphere or adding an external oxidant had adverse effects on the reaction. The protocol was amenable to aryl terminal alkynes, whereas alkyl-based terminal alkynes failed to react [57].

**Scheme 3:**
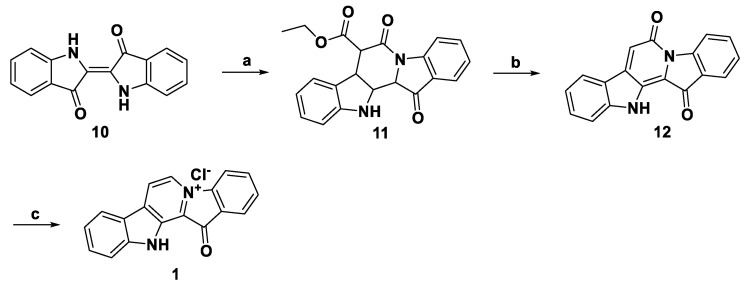
Synthetic method 5. Reagents and conditions: (a) RCH_2_COOEt, NaH, DMF, 0.5 h; (b) 40% HBr, 2 h; (c) BH_3_·THF, THF, Ar, 2 h, then H_2_O/H^+^, air, 2 h.

Zhidkov et al. reported a simple method for the synthesis of marine alkaloids 6-oxofascaplysin and fascaplysin by reaction of indigo (**10**) with active methylene compounds. The work began with the optimization of the preparation. In preliminary experiments, sodium hydroxide was replaced by sodium hydride and DMF was substituted for nitrobenzene. For subsequent hydrolysis and decarboxylation reactions, compound **11** was heated for 2 h in 40% hydrobrominated acid with reflux. The obtained compound **12** had the same spectral characteristics as natural 6-oxofascaplysin. The overall yield of the target compound was 70%. Replacement with the more selective BH_3_·THF complex in THF at reflux for 2 h, followed by hydrolysis and air oxidation, resulted in 43% yield separation (Scheme 3) [58]. 

**Scheme 4:**
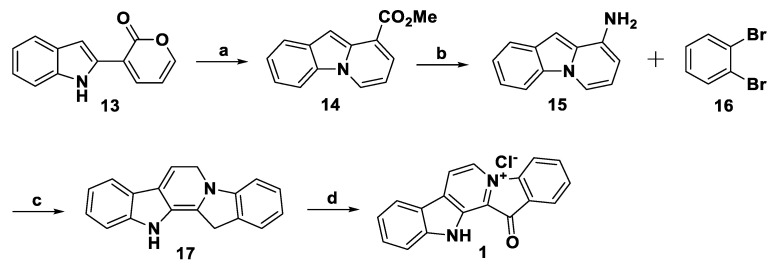
Synthetic method 6. Reagents and conditions: (a) NaOMe, CH_2_Cl_2_, MeOH (0.05 M), 23 ℃, 25 min; (b) KOH (99%), DPPA, H_2_O (94%); (c) Pd(OAc)_2_, dppf, *t*-BuONa; (d) CH_3_CO_3_H, MeOH, AcOH, conc. HCl, 0 ℃, 45 min, 85%.

A cyclization strategy can also be used to synthesize fascaplysin (Scheme 4) [59,60]. Synthesis of **14** based on indole pyranone (**13**) and sodium methoxide. Hydrolyzing ester **14** was used to provide the intermediate carboxylic acid and successfully rearranged by Curtius to furnish amine **15**. Coupling of **15** and 1,2-dibromobenzene (**16**) provided the dissociated pentacyclic-core of fascaplysin.

## 4. Structural Modification

To investigate possible therapeutic usefulness, many derivatives were designed and synthesized. We divided these derivatives into major categories according to the tested activity, and then subdivided derivatives according to the modified site.

### 4.1. Anti-Tumor Fascaplysin Derivatives

#### 4.1.1. One-Ring-Modified Fascaplysin Derivatives

**Figure 3:**
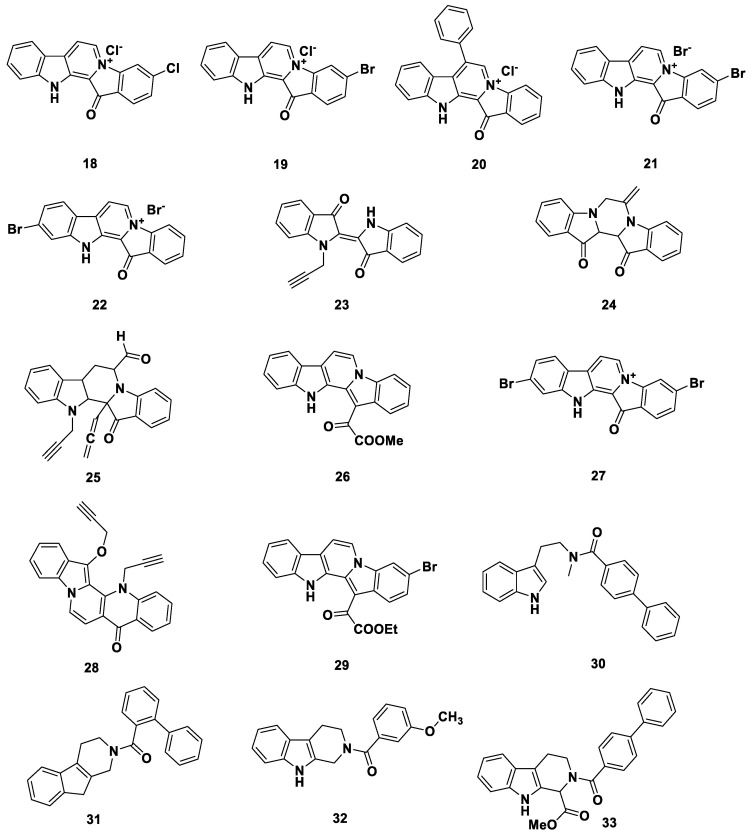
Chemical structures of and compounds **18**–**33**.

Compared with fascaplysin, 3-chloro fascaplysin (**18**, 3-CF, Figure 3) adds a chlorine atom to the E ring in the presence of chloride ions. In HUVEC and triple-negative breast cancer MDA-MB-231 cells, Bhushan et al. revealed that 3-CF simultaneously targeted diverse cancer and angiogenesis dynamics, including proliferation, chemotaxis cell migration, invasion, growth factors signaling cascade, autophagy, and apoptosis. 3-CF was more sensitive toward MDA-MB-231 cells with IC_50_ values of 0.3 μM. Furthermore, their outcomes proved that 3-CF not only induced autophagy but also apoptosis [61]. Lyakhova et al. also found that the cytotoxic efficiency of compound **18** was higher than that of unsubstituted fascaplysin [62].

Based on fascaplysin skeleton, 3-bromofascaplysin (**19**, Figure 3) is a derivative that adds a bromine atom to the E ring. Zhidkov et al. found that compound **19** showed selective cytotoxic effects on different cell lines, with the highest efficacy in melanoma cells Sk-Mel-28 [63]. The compound **19** proved to be the most effective substance for the elimination of C6 glioma cells. The number of living cells in the G0 phase was the lowest after treatment with **19**. On the U-87 MG cell line, the cytotoxic effect of **19** was superior to that of fascaplysin [64]. 

Lyakhova et al. compared compounds **20**–**22** (Figure 3) on C6 cells in vitro. Compound **20** added a benzene ring to the C ring, **21** and **22** added a bromine atom to the E ring and A ring, respectively, in the presence of bromide ions. 7-phenylfascaplysin (**20**) and 3-bromofascaplysin (**21**) showed the strongest antiproliferative activity. By the end of the experiment under the effects of **20** and **21**, the number of tumor cells was reduced to a minimum in the G0 phase [62].

#### 4.1.2. Two-Rings-Modified Fascaplysin Derivatives

Keller et al. synthesized four fascaplysin analogues **23**–**26** (Figure 3). Compounds **23**–**25** are modifications to the B and C rings. These compounds showed notable activity against lung cancer NCI-H187 and KB-oral cavity cell lines. Compound **25** showed toxicity to normal mammalian cells [65]. The C and D rings are modified in compound **26** (Figure 3). Zhidkov et al. indicated that preliminary in vitro bioassays for **26** showed the effect of inducing cell death in C6 cells. The fraction of live cells after 48 h of observation of compound **26** was 84.0% (1 μM) and 68.2% (1.5 μM). While the results of fascaplysin were 77.7% (1 μM) and 67.4% (1.5 μM) under the same conditions [66].

DBF (**27**, Figure 3) is a derivative modified in the A and E ring. In human prostate cancer cells, DBF induced apoptosis at low micro- and nanomolar concentrations and JNK1/2 was identified as one of the molecular targets of DBF. Inhibition of JNK1/2 and p38 by specific inhibitors increased the cytotoxic activity of DBF, whereas active ERK1/2 was identified to be important for the cytotoxicity of the alkaloid. Remarkably, DBF strongly synergized with Olaparib because of the induction of ROS production, and with other clinically approved platinum and taxane agents [67]. Spirin et al. found that DBF effectively inhibited the leukemia cell (K562-chronic myeloid leukemia cell line; MV4;11-acute myeloid leukemia cell line, bearing mutant *FLT3*; U937 cells) growth in nanomolar concentrations and induced apoptosis. DBF was shown to affect the S/G2 phase of cell cycle when added to all tested cell lines [68]. A study by Zhidkov et al. showed that DBF exhibited the highest selectivity to cancer cells, with SI = 7–9 [27].

#### 4.1.3. Three-Rings-Modified Fascaplysin Derivatives

For fascaplysin derivatives with modifications to three rings, there are mainly two combinations, B, D, C ring and cyclic C, D, E ring. Compound **28** was modified at the B, C, and D rings, and compounds **29**–**33** at the C, D, and E rings (Figure 3). Compound **28** showed notable activity against the lung cancer NCI-H187 and KB-oral cavity cell lines, which was equivalent to the positive control ellipticine [65]. Compound **29** induced cell death in C6 cells. The fraction of live cells after 48 h incubation of **29** was 84.5% (1 μM) and 69.3% (1.5 μM). The results of fascaplysin were 77.7% (1 μM) and 67.4% (1.5 μM) under the same conditions [66].

Chaudhuri et al. synthesized biphenyl-4-carboxylic acid-[2-(1*H*-indol-3-yl)-ethyl]-methylamide (CA224) (**30**, Figure 3), a non-planar analog of fascaplysin that specifically inhibited CDK4-cyclin D1 in vitro. CA224 was tested in a panel of ten different cancer cell lines (LS174T, PC-3, MiaPaCa, A549, Calu-1, NCI-H460, NCI-H1299, BNL CL2, BNL SV A.8) and induced growth inhibition in vitro at low micromolar concentrations. The proteins p53, p21, and p27 were up-regulated in p53-positive cancer cells (A549 and LS174T) treated with CA224, while cyclin B1 and CDK1 were down-regulated. In lung cancer cells, CA224 reduced colony formation efficiency and specifically triggered death in SV40 large T-antigen transformed cells. In SCID (severe-combined immunodeficient) mice models, it proved effective at 1/10th the MTD against HCT-116 and NCI-H460 cells xenograft. The potential for the clinical development of CA224 was suggested by its encouraging performance in human xenograft models with an excellent therapeutic window [69]. It maintained a G0/G1 block in synchronized cancer cells and prevented CDK4-specific phosphorylation of the retinoblastoma protein [70].

Chaudhuri et al. found that *N*-(biphenyl-2-yl) tryptoline (BPT) (**31**, Figure 3) was identified as a potent inhibitor of cancer cell growth (LS174T, PC-3, MiaPaCa, A549, Calu-1, NCI-H460, NCI-H1299, NCI-358, BNL CL2, BNL SV A.8) and free from DNA-binding properties owing to its non-planar structure. It demonstrated significantly stronger in vitro suppression of the CDK4-cyclin D1 enzyme than many other CDK family members. Even while it prevented cancer cells without the mitotic-spindle checkpoint from proliferating at the G0/G1 phase of the cell cycle, the block mainly occurred at the G2/M phase. BPT also inhibited tubulin polymerization and promoted tubulin depolymerization of paclitaxel-stabilized tubulin. BPT up-regulated the expression of p53, p21, and p27 while down-regulated the expression of cyclin B1 and CDK1 in p53-positive cells. BPT was reported to be effective against HCT-116 and NCI-H460 cells-derived xenograft in SCID mice models at 1/10th the maximum-tolerated dose (1000 mg/kg). Compared to fascaplysin, BPT showed uncommon capacity to prevent two overlapping but essential cell cycle phases, mitosis and G0/G1, making it a substantially more effective anticancer compound [71].

Chaudhuri et al. found that compound CA199 (**32**, Figure 3) was at least 25-fold specificity toward CDK4-cyclin D1 (CDK4-cyclin D1 IC_50_ = 20 μM, CDK2 > 500 μM). It prevented asynchronous cell development at G0/G1 in a retinoblastoma protein (pRb)-dependent way. Furthermore, in synchronized cells that had been liberated from a mimosine-induced G1/S block, CA199 only inhibited proliferation at early G1 phase [72]. Zhang et al. noted that compound **33** (Figure 3) had the strongest inhibitory activity against Hela cells (IC_50_ = 1.03 μM). The comparatively low cytotoxicity of compound **33** to normal cell lines (IC_50_ ranged from 99.82 μM to 429.00 μM) further suggested that such non-planar compounds were less selective for the normal cells and boost their safety. Additionally, the results of kinase inhibition assays mostly agreed with those of the MTT assay, and compound **33** had the strongest inhibitory effects against CDK4 of all the synthetic compounds [73].

#### 4.1.4. Four-Rings-Modified Fascaplysin Derivatives

**Figure 4:**
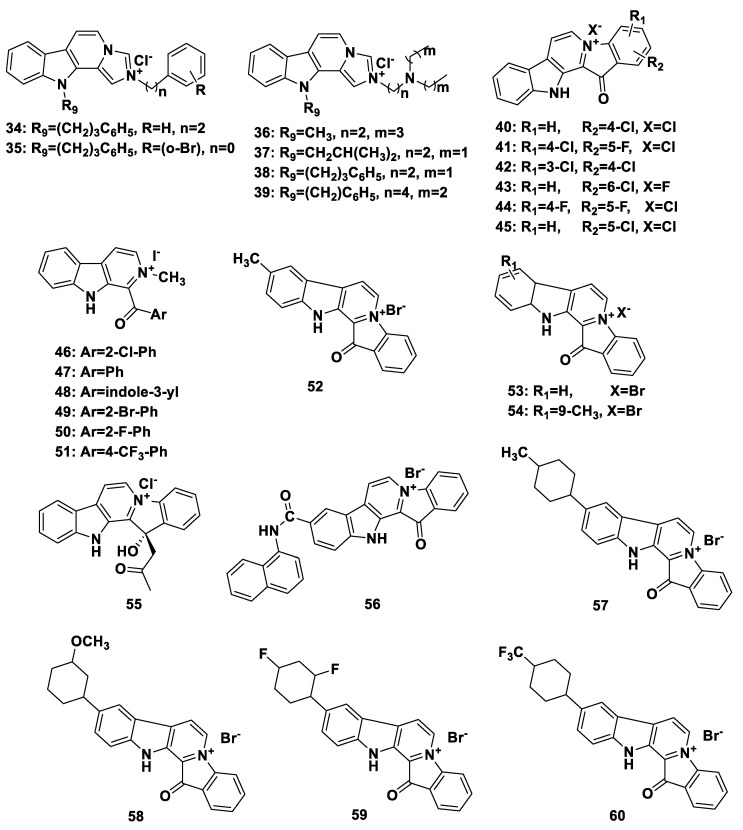
Chemical structures of compounds **34**–**60**.

All four ring modifications of fascaplysin are concentrated on the B, C, D, and E rings. Guo et al. synthesized several fascaplysin analogs; these compounds (**34**–**39**, Figure 4) had strong inhibitory effects on cancer cells (A549, BGC-823, CT-26, Bel-7402) with IC_50_ values lower than 10 μM. Additionally, in vitro VEGFR2 inhibitory properties of high-efficiency compounds were assessed. The 3-phenylpropyl substituent at the *N*^9^-position of the indole ring was found to be the most effective group for ensuring potent cytotoxic effects, and the amino side chain groups were found to be helpful pharmacophoric groups for enhancing the antiproliferative activity, according to a structure–activity relationship (SAR) analysis. Compound **39** might significantly and dose-dependently prevent tube formation in EA.HY926 cells, according to early research on its mechanisms of action [74].

### 4.2. Anti-Alzheimer Fascaplysin Derivatives

Compounds **40**–**45** were obtained by modifying the C, D, and E rings of fascaplysin. Compounds **46**–**51** were obtained by modifying the C and D rings of fascaplysin. Bharate et al. pursued the medicinal chemistry of fascaplysin to establish its SAR for P-gp induction activity. Only quaternary nitrogen-containing analogs **40**–**45** and **46**–**51** showed promising P-gp induction activity after a series of substituted quaternary fascaplysin analogs were synthesized and screened for P-gp induction. P-gp expression was 4–8 fold higher after human colon adenocarcinoma LS-180 cells were incubated with **44** and **49** at 1 μM. AChE, an enzyme that caused neuronal death in Alzheimer’s disease, was also inhibited by compound **44**. Results suggested that this scaffold had the potential for the use in the creation of anti-Alzheimer agents [50].

Compounds **52**–**54** were obtained by modifying the A ring. 9-methylfascaplysin, **52**, was more potent than fascaplysin to inhibit Aβ-fibril formation. Compound **52** was also found to prevent the formation of Aβ oligomer in vitro. In addition, **52** at low concentrations protected human neuroblastoma SH-SY5Y cells from neurotoxicity of Aβ oligomer in vitro [75]. Cui et al. studied the in vitro toxicities, cholinesterase inhibition activities and neuronal protective effects of several derivatives. The effects of compounds **53** and **54** on animal cognition were further examined. Both **53** and **54** ameliorated the cognitive impairment brought by scopolamine or Aβ oligomers in mice without compromising their ability to move around. Additionally, they discovered that **53** and **54** reduced cholinergic dysfunctions, decreased pro-inflammatory cytokines expression, and inhibited Aβ-induced tau hyper-phosphorylation in vivo. The most significant finding was that **54** appeared to cross the blood–brain barrier and remained in the central nervous system. All these results indicated that fascaplysin derivatives were effective multi-target AD inhibitors and would have therapeutic applications for AD treatment [76].

### 4.3. Antiplasmodial Fascaplysin Derivatives

Four fascaplysin analogs (**23**–**26**, Figure 3) were designed by Kelleri and colleagues. All four compounds showed notable antiplasmodial activity. Furthermore, compounds **23** (antiplasmodial IC_50_ = 1.1 μM) and **24** (antiplasmodial IC_50_ = 0.85 μM) were more effective than compounds **25** and **26** [66]. Compound **55** (homofascaplysin A) is the compound obtained by modifying the D ring. Van Wagoner et al. assessed the activities of **55** against different blood-borne life stages of the malaria pathogen plasmodium falciparum. Compound **55** showed an IC_50_ of 0.55 nM against ring stage parasites and 105 nM against all live parasites. Given the stronger resistance of ring-stage parasites against most current antimalarials relative to the other blood stages, **55** represented a promising agent for the treatment of drug-resistant malaria [77].

### 4.4. Antibacterial Fascaplysin Derivatives

Compound **56** is a ring modification compound on the basis of fascaplysin. Liang et al. found that **56** was the most potent compound against Methicillin-resistant *Staphylococcus aureus* (MRSA) with an MIC value of 0.098 mg/mL (10-fold lower than vancomycin). Subsequent mechanisms exploration indicated that **56** had a relatively stronger ability to destroy bacterial cell walls and membranes, as well as a higher binding affinity to bacterial genomic DNA. Compound **56** could inhibit MRSA biofilm formation in vitro and bacterial infection in vivo. All these results illustrated that **56** was a strong and safe multi-target antibacterial agent, which made it an attractive candidate for the treatment of MRSA and its biofilm infections [78].

Liang et al. proposed a two-step reaction sequence of regioselective Suzuki–Miyaura cross coupling and intramolecular quaternization to build a family of fascaplysin derivatives. It was also found that the synthesized derivatives have good antibacterial activity. Fascaplysin as positive controls for Gram-positive bacteria MRSA (ATCC 43300) and Gram-negative bacteria *Escherichia coli* (ATCC 25922), the MIC values are 0.78 μg/mL and 12.5 μg/mL, respectively. Compounds **57**–**60** showed an enhanced anti-MRSA activity. Therein, compound **59** showed the best antibacterial activity against MRSA (ATCC 43300) with an MIC value of 0.20 μg/mL, which was four-times more potent than fascaplysin. Moreover, compounds **59** and **60** showed the best antibacterial activity against *E. coli* (ATCC 25922) with MIC values of 1.56 μg/mL, which were eight-times more active than fascaplysin [79].

The following table (Table 1) summarizes the IC_50_ values of related compounds.

## 5. Conclusions

Fascaplysin is a very promising marine-derived natural product with several biological activities. Fascaplysin and its derivatives effectively inhibit the growth of tumor cells. Moreover, it also exhibited potent anti-plasmodium activity, antimicrobial activity, anti-oxidize activity, anti-inflammatory activity, and so on, which points to a new direction for the further development of fascaplysin. At present, many excellent studies about fascaplysin have been published, but related research still faces both challenges and unmet opportunities. Due to the special planar structure of fascaplysin, it can be embedded in DNA, which is highly toxic and may have a certain impact on its drug product. In the future, to reduce the toxic impact, in addition to the structural modification of the compound, the development of corresponding targeted drugs are also possible to make its orientation come into play. These promising biological activities associated with fascaplysin have already led to the discovery of a few synthetic lead molecules, and there is much unexplored medicinal chemistry space that may further lead to the discovery of novel lead compounds.

## Figures and Tables

**Table 1:** IC_50_ values of fascaplysin and some derivatives.

Compound	Target	IC_50_ Value
Fascaplysin	HL-60	0.5 μM
	Sk-Mel-28	0.03–0.22 μM
	SCLC	0.89 μM
	CTCs	0.57 μM
	NSCLC	1.15 μM
	LNCaP	0.54 μM
	AChE	1.49 μM
	Hela	0.56 ± 0.05 μM
	*P. falciparum* strain K1	50 ng/mL
	*P. falciparum* strain NF54	34 ng/mL
Compound **18**	MDA-MB-231	0.3 μM
Compound **19**	Sk-Mel-28	1.1–1.9 μM
Compound **23**	antiplasmodial	1.1 μM
Compound **24**	antiplasmodial	0.85 μM
Compound **30**	CDK4-D1	5.5 μM
Compound **32**	CDK4-D1	20 μM
	CKD2	>500 μM
Compound **33**	Hela	1.03 ± 0.19 μM
	normal cell lines	99.82 ± 9.97–429.00 ± 38.87 μM
Compound **55**	ring stage parasites	0.55 ± 0.11 nM
	all live parasites	105 ± 38 nM
